# Benefits and risks of using bacterial- and plant-produced nano-silver for Japanese quail hatching-egg sanitation

**DOI:** 10.1007/s00203-023-03547-3

**Published:** 2023-05-09

**Authors:** Nagwa H. Hamouda, W. D. Saleh, N. F. Nasr, M. I. El Sabry

**Affiliations:** 1 Department of Microbiology, Faculty of Agriculture, Cairo University, Giza, 12613 Egypt; 2 Department of Animal Production, Faculty of Agriculture, Cairo University, Giza, 12613 Egypt

**Keywords:** Nano-silver, Microbial load, Hatchability, Sanitation, Poultry, Chick quality

## Abstract

This research compared how bacterial-, plant-produced silver nanoparticles (Ag-NPs) and TH4 affected the eggshells microbial load and quail chicks' liver structure, embryonic mortality, and features related to hatchability. Ag-NPs were sensitized by bacterial and plant methods, and then identified by UV–visible spectroscopy, TEM, and FTIR spectroscopy. B-Ag-NPs were found in spherical shapes in size ranging from 7.09 to 18.1 nm versus multi-shape with size range of 25.0–78.1 nm for P-Ag-NPs. A total number of 624 eggs (in three equal groups) of Japanese quail flock were sprayed with TH4 as control, B-Ag-NPs and P-Ag-NPs. Thereafter, three eggs were sampled randomly from each group for determining important microbial groups. The remaining eggs were incubated according to the recommended incubation conditions. On the day of hatching, the percentages of hatchability and embryonic mortality were measured. Besides, five chicks from each treatment were slaughtered and the livers were utilized for ICP and histological tests. The effects of all three treatments on the microbial count in eggshells were comparable, according to the results. In addition, there was no negative effect on either hatchability percentage or embryonic mortality rate. The liver structure from both B-Ag-NPs and P-Ag-NPs treatments exhibited severe and moderate degeneration of hepatocytes, which may indicate possible hazardous effects of using nanoparticles. Using TH4 did not cause liver structure abnormality. In conclusion, using Ag-NPs for sanitizing hatching eggs effectively reduces the eggshell microbial count without affecting the hatchability percentage. Nevertheless, histological changes are appropriate to be considered as a safety parameter in Ag-NPs applications.

## Introduction

Poultry production is growing annually to meet the human demands for protein. Quail (*Coturnix coturnix*) is one of the popular poultry types, which spread over Northern Africa, Europe and Asia (Puigcerver et al. 2012; El Sabry et al. 2017). Quail production is expected to increase due to the fast growth and high demand of the birds in several countries such as Japan, Ukraine, Canada, and Russia (Katerynych and Pankova 2020).

However, the infectious diseases are among of the real challenges that hinder the improvement of poultry industry (El-Sabry et al. 2012; Mehaisen et al. 2016; Parrott and Walley 2017). The infection of hatching egg occurs at the end of egg tract, that caused to the contact of eggshell with feces or bedding after oviposition and/or during handling and transportation of eggs. Bacteria*,* yeasts and molds are typical contaminants found on the surfaces of eggshell. The presence of many different of pathogenic microorganisms, *e.g., Staphylococcus aureus, Salmonella* spp., *Escherichia coli*, molds and yeasts on eggshell surfaces represents a possible threat contamination of the contents of the eggs (De et al. 2004; Singh et al. 2009; Aygun et al. 2012; Jones et al. 2012). Pathogenic microorganisms, which penetrate the eggshell finally cause low proportion of hatchability and poor-quality chicks. Thus, the most critical point to control the quality of hatchery eggs is sanitation to obtain healthy and high-quality chicks using efficient disinfectants (Ibrahim et al. 2018a).

Nanotechnology can be a part of the solution to restrict the spread of bacterial diseases through the poultry industry chain. Nano-silver has shown potential to be an efficient disinfectant (Hamouda et al. 2023) due to the tiny size of silver particles grant them strong bacteriostatic and bactericidal effects on a broad spectrum of microorganisms (Panyala et al. 2008; Chmielowiec-Korzeniowska et al. 2015; Ibrahim et al. 2018a). For instance, Li et al. (2010) found that exposing *E. coli* cells to various nano-silver concentrations (10 and 50 μg/ml) perforated the bacterial cell membrane and decreased the activity of some membranous enzymes, which eventually cause the death of *E. coli* bacteria. Nevertheless, Loghman et al. (2012) showed harmful effects of high doses of nano-silver (8 and 12 ppm) on the liver cells in broilers, which raise some fears about its toxicity effects on the embryos inside the eggs. Thus, nano-silver might be utilized as a disinfectant in the poultry industry only with caution to avoid toxic hazards in birds (El-Sabry et al. 2018; Hamouda et al. 2023).

Despite the potentiality effects of silver nanoparticles (Ag-NPs) as antimicrobial agents have been shown, a few studies had been compared between the effects of Ag-NPs that synthesized by different methods. The plant and bacterial methods as new trends in nano-material manufacturing open the door for enhancing the safety of nano-products (El-Sabry et al. 2018; El Sabry et al. 2021). Therefore, the objective of the current study was to assess the effects of bacteria- and plant-synthesized Ag-NPs on the eggshells microbial Load. Also, the hatchability percentage, embryonic mortality and histological structure of the liver were investigated to assess the effects of Ag-NPs on the hatching traits.

## Experimental

### Animal and ethical statement

This protocol was approved by Cairo University Ethics Committee for the Care and Use of Experimental Animals in Education and Scientific Research (Protocol number: CU-II-F-12-22).

### Preparation and characterization of bacteria- and plant-synthesized silver nanoparticles

#### Bacterial extracellular synthesis of silver nanoparticles (B-Ag-NPs)

Media were prepared, sterilized, and inoculated with a fresh subcultured of the strain *Pseudomonas aeruginosa* ATCC 35,032. The inoculated flasks were cultured in a shaking incubator at 30 °C for 48 h, after that the culture was centrifuged at 10,000 rpm for 15 min. Biosynthesis of Ag-NPs was done using the supernatant. The preparation of AgNO_3_ stock (1 M) was sterilized by filtration 0.2 µm filter. The silver nitrate (9 ml of conc. 1 mM) was combined with the separated bacterial supernatant (1 ml) in a sterilized Erlenmeyer flask to reach a final concentration of approximately 1 m (0.9 mM). The reaction between these supernatants and Ag^+^ was done in light conditions for time intervals of 30 min, 1 h (Ibrahim et al. 2018b).

### Plant synthesis of silver nanoparticle (P-Ag-NPs)

Five ml of mint (*Mentha piperita*) leaves extract was mixed with 100 ml of AgNO_3_ solution (1 mM). The mixture was heated at 90 °C for 1 h, with constant stirring of 500 rpm, until the color changed from pale green to dark brown and then stored under darkness at room temperature for 24 h to test the stability of Ag-NPs (Gabriela et al. 2017; Hamouda et al. 2023).

### Characterization of Ag-NPs

#### UV–Vis spectral analysis

Spectral analysis for the synthesis of Ag-NPs was analyzed by ultraviolet–visible spectroscopy using UV–Vis Spectrophotometer (Cary100, Japan) in the range from 200 to 800 nm.

### Fourier transforms infrared (FTIR) spectroscopy analysis

Profiling of Ag-NPs functional groups was identified using FTIR spectrometer (Infrared Spectrum Origin JASCO FT/IR-6100 type-A, Japan). Vacuum-dried Ag-NPs were characterized in the range 400–4000 cm^−1^ at a resolution of 4 cm^−1^ using potassium bromide (KBr) pellet method. (Basavaraja et al. 2008; Velmurugan et al. 2015; Faghihzadeh et al. 2016; Karthik et al. 2020).

### Transmission electron microscopy (TEM)

The size and distribution of Ag-NPs particle were measured using TEM (JEOL model JEM-2011) at 80 kV as accelerating voltage. The samples were spread (5 µl) over copper grids coated with carbon, which were then dried in a silica-filled desiccator (Hamouda et al. 2023).

### Antimicrobial activity of biosynthesized Ag-NPs

The antimicrobial activity of biosynthesized B-Ag-NPs was assessed against the following target microorganisms: Gram-positive bacteria; *Bacillus cereus* ATCC 33,018, *B. subtilis* ATCC 6633, and *Staphylococcus aureus* ATCC 25,923; methicillin-resistant *Staph. aureus* (MRSA) 43,300 ATCC. MRSA was kindly provided from Naval Medical Research Unit 3 (NAMRU-3). Their efficacy was tested against Gram-negative bacteria; *E. coli* ATCC 35,218, *E. coli* O157 ATCC 700,728; *P. aeruginosa* ATCC 35,032 and *S. typhimurium* ATCC 14,028, in addition to the filamentous fungus *Aspergillus niger* NRRL 1957, and the yeast *Candida albicans* ATCC 10,231. The tested microbial cultures were cultivated and tested on Mueller Hinton (MH) agar (Atlas 2006) for bacteria and MH agar supplemented with 2% glucose for yeast and fungi. The well-diffusion method was used to assess the activity of Ag-NPs as antimicrobial agent using the pour plate method and the same culture media. A 20 μl of synthesized Ag-NPs was added to the wells. Bacterial plates were incubated at 30 °C for 24 h and fungi for 72 h (Alastruey-Izquierdo et al. 2015; Feroze et al. 2020; Huq 2020). Similar test for the antimicrobial activity spectrum of P-Ag-NPs was previously published by Hamouda et al. (2023).

### Treatment of eggs with biosynthesized silver nanoparticles

An experimented group was sprayed with B-Ag-NPs, while the other was sprayed with P-Ag-NPs. A total number of 624 eggs was classified as follows; 208 eggs were sorted in 13 racks for treatment with silver nanoparticles based on bacterial extract, 208 eggs were sorted in 13 racks for plant-based silver nanoparticles, 13 racks containing 208 eggs treated with TH4 (as a commercial disinfectant containing didecyl dimethyl ammonium chloride, dioctyl dimethyl ammonium chloride, and octyl decyl dimethyl ammonium chloride as active ingredients). All samples were evaluated to the microbiological examinations to determine total bacterial count, total fungi, total spore-forming bacteria, Enterobacteria, total and fecal coliforms, and detection of some pathogenic species such as *Salmonella* sp. Three eggs of each treatment were soaked in 50 ml of saline solution, and serial dilutions were prepared. Sampling was conducted for untreated eggs at 1 h, 7 days, and 15 days of spraying. For histopathology, chick samples were taken at 18th day, directly after hatching to examine the existence of Ag-NPs in the chick tissues.

### Microbial load determination on the eggshell

Each sampled egg from each treatment was aseptically collected in a sterile plastic bag containing 50 ml of sterile saline (0.9% NaCl). Eggs were gently massaged for one minute and then removed. The washing water was tenfold diluted and 0.1 ml of each dilution was incubated on Mueller Hinton (MH) agar (Atlas 2006) for bacteria and MH agar supplemented with 2% glucose for yeast and fungi (Willinghan et al. 1996), and incubation took place at 37 °C for 48 h, then bacterial counts were monitored. Microbial loads were expressed as colony forming units (CFU) per ml of sample. Isolation and identification of developed colonies were conducted according to Holt et al. (1994).

### Hatching conditions

During the first 15 days of incubation, eggs from all treatments hatched at a standard temperature of 37.5 °C and 52% relative humidity (RH). Then, eggs were kept in hatcher baskets for the last 2 days, at 36.5 °C and 65% RH. All the chicks were counted and weighed within 45 min of hatching. Hatchability percent (of fertile eggs) was calculated and the unhatched eggs were broken out. The number of infertile, early mortality (from day 1 to day 7 of incubation period), middle mortality (from day 8 to day 14 of incubation period), and late mortality (from day 15 to day of hatch) were recorded. Then, the percentages of early, mid and late embryonic mortalities were calculated. According to Ibrahim et al. (2018a), quality of the quail chick was determined to categorize chicks as either A or B grade. If chick has one of the following: unhealed navel, leg abnormalities or too weak to stand, dirty or other abnormality, it was categorized as B-grade chicks.

### Inductively coupled plasma analysis (ICP)

Tissue samples from the breast muscle and liver were collected for analysis of elemental composition using ICP analysis. Liver samples were subjected to acid digestion using a microwave digestion system (Multiwave PRO, Anton-Paar) with 5 mL of 65% HNO_3_ as the acid reagent. The determination of silver was carried out using an Agilent 5100 Synchronous Vertical Dual View (SVDV) ICP-OES equipped with Agilent Vapor Generation Accessory VGA 77. The samples were digested to ensure a suitable matrix for measuring Ag^+^ and to provide acceptable and consistent recovery compatible with the analytical method as described in the guidelines (APHA AWWA 2017). An intensity calibration curve was constructed for each series of measurements using a blank and three or more standards from Merck Company (Germany). Accuracy and precision of silver ion measurements were validated using external standards reference from Merck.

### Histopathological investigation of liver

Five samples of the liver were collected from newly hatched chicks of each treatment. Samples were fixed in Bouin solution and stained with hematoxylin and eosin. The histological examination was done using three serial sections from the liver using a light microscope (XSZ-PW 146-Proway Optics and Electronics, China) at a magnification power of 40 × (El Sabry et al. 2015).

### Statistical analysis

All assumptions were tested using Shapiro–Wilk test for normality. Analyses were performed using JMP Pro 5 statistical analysis program. One-way ANOVA was used to analyze the treatment effect on hatchability, chick quality traits, stress indicator organs’ weight and embryonic mortality.

## Results and discussion

### UV–Vis spectroscopy

**Fig. 1:**
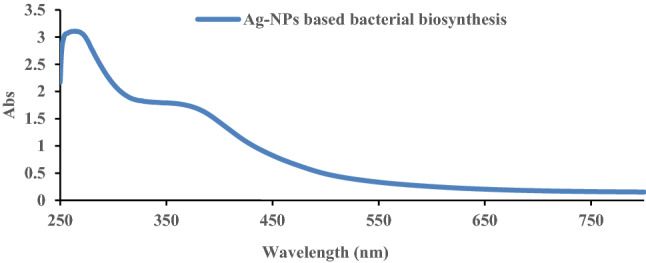
UV–Vis spectra of Ag-NPs synthesis of B-Ag-NPs; bacterial biosynthesis method

The biosynthesis of Ag-NPs can be verified by determined the surface plasmon resonance (SPR) band by the UV–Visible spectra of synthesized nanoparticles. The spectrum of UV–Vis represents a qualitative idea about the nanoparticles' size and shape, these results can be predicted from the width, shape, and position of the SPR band (Huang et al. 2011; Riaz et al. 2021). Figure 1 shows a wide peak observed at 380 nm, which is a defining band for the Ag-NPs formed by bacteria-based biosynthesizing method. The shoulder at 380 nm following this SPR peak indicates the existence of spherical shaped nanoparticles in the solution (Hamouda et al. 2023). Similar results were also investigated by Riaz et al. (2021) and Agnihotri et al. (2014); they found the same peak at 420 nm wavelength for spherical Ag-NPs with size of 50 nm.

Mint leaves extract, rich in phytochemicals including polyphenols, can act as a bioreductive and stabilizing agent for Ag-NPs. The findings by Gabriela et al (2017) reported that the extract contains various phytochemicals, including polyphenols, which have the ability to reduce Ag + to Ag0 and bind metallic ions into nanoparticles. The functional groups responsible for this activity were hydroxyl, carbonyl, and carboxyl. Further research is needed to explore its applications and potential environmental impact. Furthermore, Shaheen (2021) and Salem (2023) reported that the biosynthesis of nanoparticles involves bio-reduction capping and trapping. Enzymes present in the cell wall of microorganisms convert metal ions and metal oxide into nanoparticles, which then diffuse away from the cell membrane. Biological molecules like sugars, carbohydrates, enzymes, and proteins act as capping and reductants. The mechanism for creating nanoparticles utilizing biological models is complex and varies depending on the biological agent.

### FTIR spectral characteristics

**Fig. 2:**
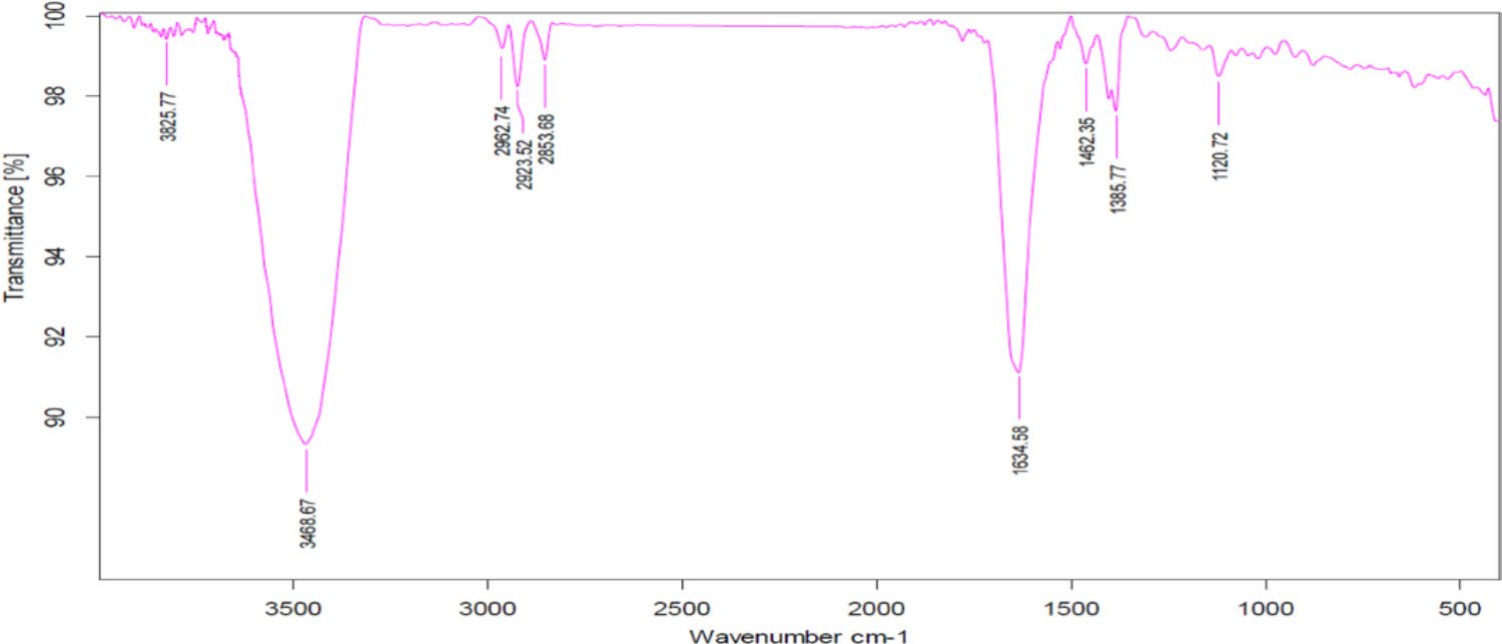
FTIR spectral characteristics of bacteria-biosynthesized Ag-NPs

The biofunctional groups of the Ag-NPs biosynthesized by bacteria were identified and are presented in Fig. 2. In light of this study and another study of the same team (Hamouda et al. 2023), both bacteria- and plant-based Ag-NPs showed the prominent peaks at 3468, 2962, 2923, 2853, 1634, 1462, 1385 and 1120 cm^−1^ with small peaks between 1100 and 500 cm^−1^, these peaks refer to the presence of vibrations of (O–H) group, that is typically assigned for the phenol and/or carboxylic group in bacterial metabolites, the stretching bond of C–H and N–H bending vibration from reducing and capping by plant extract at the bands 2962, 2923, and 2853 cm^−1^, stretching of C = O bonding at 1634 cm^−1^, symmetrical stretching for N–O group of nitro compounds at 1385 cm^−1^. The elemental (sulfur or phosphorus) function group presented at 1120 cm^−1^, and the bending region of the aliphatic chain showed at the peaks between 1100 and 500 cm^−1^, respectively.

Similar results reported the exitance of this band at nearly the same position; 3441.01 cm^−1^ (Nithya-Deva-Krupa and Raghavan 2014), 3401 cm^−1^ (Sadeghi and Gholamhoseinpoor 2015), and the absorbance bands around 3427–3436 cm^−1^ (Hamouda et al. 2019). In addition to the stretching bond of C–H showed at the bands 2986, 2944, 2852 cm^−1^, this concurs with Hamouda et al. (2019). They reported the presence of peaks at 2924, 2854 and 1455 cm^−1^ and attributed them to aliphatic C–H stretching vibration of hydrocarbon chains and N–H bending vibration. Additionally, Gabriela et al (2017) have proven the hydroxyl, carbonyl, and carboxyl functional groups present in various phytochemicals in mint leaf extracts are responsible for the bio-reduction and stabilization of silver nanoparticles (AgNPs). *M. piperita*, which is abundant in polyphenols, including tannic acid, can reduce Ag + into Ag0 and bind metallic ions into nanoparticles. The high polyphenolic content of up to 19% in *M. piperita* makes it an excellent source of bioreductive and stabilizing agents for AgNP's.

### Transmission electron microscopy (TEM)

**Fig. 3:**
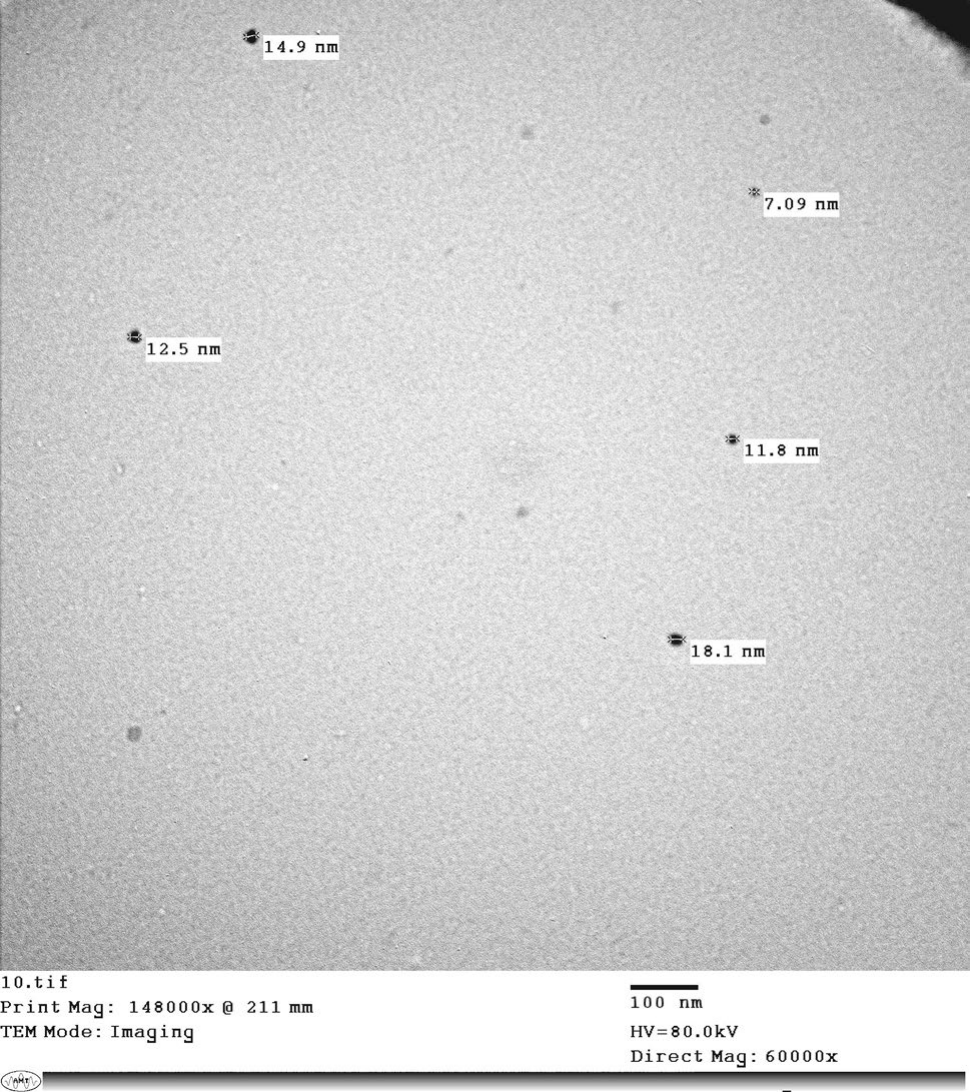
TEM for the bacterial synthesized Ag-NPs

The TEM investigation identified the shape and size of synthesized Ag-NPs. The B-Ag-NPs had spherical shape and a particle size range of 7.09–18.1 nm (Fig. 3), whereas Hamouda et al. (2023) found that the plant-synthesized Ag-NPs (P-Ag-NPs) were of multi-shape spherical, triangular, or irregular with size range of 25.0–87.1 nm.

The variation in shape and size between bacterial and plant Ag-NPs may be attributed to the difference in enzymes, organic acids, and biosurfactants present in the supernatants, which showed various reducing, chelating and binding characteristics. Also, Gabriela et al (2017) reported the use of *M. piperita* leaf extract for the green synthesis of Ag-NPs can diversify range of shapes (triangular, spherical, and irregular morphologies), with a lower mean size of 50 nm compared to other studies.

### The antimicrobial activity of biosynthesized Ag-NPs

The results in Table 1 show that all tasted bacterial and fungal strains were inhibited using B-Ag-NPs with zone of inhibition ranged from 10 mm (for *Aspergillus niger*) to 21.5 (for *Staphylococcus Aureus*); bacterial strains were more sensitive to B-Ag-NPs than fungal strains.

**Table 1:** Antimicrobial activities of bio-Ag-NPs against studied G− and G + bacterial and fungal strains (Inhibition zone, mm ± SD)

	*Escherichia coli*	*Escherichia coli O157*	*Staphylococcus aureus*	*Staph. aureus (MRSA)*	*Salmonella typhimurium*	*Bacillus subtilis*	*Bacillus cereus*	*Pseudomonas aeruginosa*	*Aspergillus niger*	*Candida albicans*
Bacterial Ag-NPs	12.5 ± 1.12	13.75 ± 0.43	21.5 ± 1.12	16.25 ± 0.83	10.75 ± 0.83	14.25 ± 1.09	14.25 ± 0.83	12.75 ± 0.77	10 ± 0.00	12 ± 1.22
Plant Ag-NPs*	12.50 ± 0.89	13.00 ± 1.10	12.00 ± 1.81	12.00 ± 0.93	13.00 ± 1.32	12.00 ± 0.87	12.67 ± 1.03	12.25 ± 0.56	11.50 ± 1.37	15.75 ± 1.67

*Results of our previous work adopted from Hamouda et al. (2023)

The same authors in a previous paper (Hamouda et al. 2023) reported that P-Ag-NPs could inhibit the tested bacterial strains with similar effect just ranged between 12 and 13 mm inhibition zone diameter, but *Candida albicans* was more sensitive to P-Ag-NPs with 15.75 mm inhibition zone diameter. In the same direction, Ibrahim et al., (2018b) found that bacterial based Ag-NPs using *Rhizobium leguminosarum* showed antimicrobial activity against pathogenic bacterial strains like *Staph. aureus,* and *B. cereus* and fungal strains such as *A. niger* and *Candida albicans*. Also, Saeed et al. (2020) recorded inhibition zone diameter ranged between 11 and 28 mm of pathogenic bacterial strains using B-Ag-NPs mediated by *E. coli*. Similar findings were reported by Mohammed et al. (2022); they found that the plant-based biosynthesized Ag-NPs by *M. oleifera* extract demonstrated a possible antibacterial activity against some pathogenic strains such as *E. coli*, *Klebsiella pneumoniae*, *B. subtilis*, and *Staph. aureus*. Also, Bindhu et al. (2020) and Prasad and Elumalai (2011) reported the same activity as antibacterial agent of the plant-based biosynthesized Ag-NPs against different pathogenic bacteria.

### Eggshell microbial disinfection

In Table 2, the total Enterobacteriaceae, and total and fecal coliforms on the eggshell were decreased by both Ag-NPs and TH4. Opposed to that, total bacteria and spore formers showed slight reduction in counts. Total bacterial counts on the eggshell of eggs treated with B-Ag-NPs, P-Ag-NPs and TH4 were 3.1 ± 0.33, 3.3 ± 0.24 and 4.23 ± 0.19 log count / one egg surface, respectively. Similar reduction pattern was recorded for total fungi with all treated eggs not detected, 0.3 ± 0.24 and 0.35 ± 0.32 log count / one egg surface, respectively. In contrast, the coliform count decreased with all treatments with Ag-NPs and was not detected at the end of the incubation period. It is worth noting that *Salmonella* was not detected during the experimental period. Interestingly, biosynsysied Ag-NPs had the same antimicrobial impact on the samples as the commercial agent (TH4). De Reu et al. (2007) reported that the natural eggshell contamination was dominated by G + *Staphylococcus spp.* and Board and Tranter (1995) elucidated that Gram-positive bacteria may have originated from dust, soil or feces and their dominance may be attributed to their tolerance to dry conditions.

**Table 2:** Microbial counts (log count/ one egg surface) on the eggshell surfaces at 0, 1 h, 7 days and 15 days after treatment with bacteria-based (B-Ag-NPs), plant-based (P-Ag-NPs) and TH4 as control

	Sampling times	Total bacterial count	Total spores	Total fungi	Enterobacteriaceae	Total coliforms	Fecal coliforms	*Salmonella*
	0	5.98 ± 0.12	2.15 ± 0.12	2.53 ± 0.03	2.3 ± 0.24	1 ± 0	1 ± 0	ND
B-Ag-NPs	1 h	4.72 ± 0.03	3.49 ± 0.27	1.23 ± 0.19	ND	ND	ND	ND
	7d	4.13 ± 0.04	3.03 ± 0.21	0.33 ± 0.47	ND	ND	ND	ND
	15d	3.1 ± 0.33	ND	ND	ND	ND	ND	ND
P-Ag-NPs	1 h	4.66 ± 0.15	2.48 ± 0.26	1 ± 0	ND	ND	ND	ND
	7d	3.67 ± 0.15	1.81 ± 0.65	ND	ND	ND	ND	ND
	15d	3.3 ± 0.24	2.1 ± 0.14	0.3 ± 0.24	ND	ND	ND	ND
TH4	1 h	5.16 ± 0.37	3.72 ± 0.08	1.35 ± 0.52	ND	ND	ND	ND
	7d	3.06 ± 0.37	3.41 ± 0.08	1.55 ± 0.52	ND	ND	ND	ND
	15d	4.23 ± 0.19	ND	0.35 ± 0.32	0.51 ± 0.73	0.79 ± 1.13	0.79 ± 1.13	ND

*ND* not detected

In the current study (Table 2), after 14 dayes of treatment with B-Ag-NPs, total count of bacteria on the eggshell decreased to 3.1 ± 0.33 and the total and fecal coliforms were not detected on eggshells. Similar findings were obtained by treating the eggs with P-Ag-NPs, where total bacterial load of eggshell decreased to log 3.3 ± 0.24 count/one egg surface. Total and fecal coliforms and *Salmonella* were not found on eggshell surfaces. Samples treated with biosynsysied Ag-NPs presented a similar antimicrobial results as the commercial agent (TH4). Previous studies had shown a great flactuation in total number of bacteria on the eggshell and hatchery equipment. The air in the poultry house, the litter, and the machine of hatchery are the sources of bacterial contamination of eggshells (Smith et al. 2000; Protais et al. 2003; De Reu et al. 2005; Kim and Kim 2010). Moreover, De Reu et al. (2006) proved that higher eggshell contamination can lead to low hatchability percentage and lower chick quality. Clean hatching eggs with minimum microbial contamination is required for successful hatching process. Board and Tranter (1995) reported a wide range in the contamination levels of hatching eggs that were between 10^2^ and 10^7^. It is well known that eggs infected with bacterial pathogenes have a big share in spreading of diseases. These dangerous bacteria can decrease the hatchability percentage, increasing early chick mortality and cuase embryonic death. Smith et al. (2000) mentioned that several variables, such as the bacteria load in the poultry facility's air, affect the load of bacterial count of eggshell. According to current research, the bacterial load in poultry houses' air is positively connected with the initial eggshell contamination by bacteria (Protais et al. 2003; De Reu et al. 2005).

In the current study, bacterial contamination (total bacterial count and total coliforms) on the eggshell disinficted with Ag-NPs was lower than that of eggs disinfected with TH_4_ (control). Moreover, use of Ag-NPs can decrease the total bacterial count and coliform-free. The Ag-NPs antimicrobial properties were previously reported by Cho et al. (2005). Lankveld et al. (2010) suggested that bacterial deactivation by Ag-NPs includes catalytic oxygenation, reactions with the bacterial cell wall, protein denaturation and bonds with the DNA. It is worth noting that microbial recovery (after 15 days) was observed for TH4 in all readings except total spores, whereas with P-Ag-NPs it was only found for total fungi and it was not existing with B-Ag-NPs.

### Hatching performance

The bacterial pathogen on the surface of egg has the possibilities to penetrate the eggshell and harm the embryo. Efficient egg disinfectants play a vital role in minimizing the bacterial count on the eggshell which positively enhance hatching performance. In the current study, the early, mid and fatality rates for late embryos of B-Ag-NPs, P- and TH4 groups were almost equal. Similarly, Ibrahim et al. (2018a) found that early, mid and late embryonic mortality rates of TH4 and Ag-NPs groups were similar. Ibrahim et al. (2014) reported that inhibition of the pathogen activity on the surface of eggshell resulted in lowering the embryonic mortality rate by 10%.

Also, current study`s findings showed that the percentage of hatchability of fertile eggs with the B-Ag-NPs, P-Ag-NPs, and TH4 groups were similar (Table 3). It seems that the type of sanitizing agent had no effect on the percentages of hatchability.

**Table 3:** Effects of disinfecting quail hatching eggs with TH4, plant- and bacteria-synthesized silver nanoparticles on hatchability and embryonic mortality percentages

Treatment	Hatchability of fertile eggs %	Embryonic mortality (%)		
		Early	Mid	Late
Control (TH4)	73.5	13.28	2.42	9.78
B-Ag-NPs	74.51	9.15	3.72	13.62
P-Ag-NPs	78.38	10.82	2.68	8.12
± MSE*	2.95	1.98	1.26	2.90
*P*-value**	0.50	0.39	0.75	0.44

*MSE mean standard error, ** *P*-value probability value

### Chick quality

The quality of chicks is an essential parameter for the assessment of the effectiveness of different treatments, including the use of nano-silver. The weight of the chick at the time of hatching is a reliable indicator of chick quality, which has been confirmed by previous studies (Willemsen et al. 2008; El Sabry et al. 2013). In this study, the weight of quail chicks on the day of hatch was similar across all groups (7.5 ± 0.5 g), indicating that the treatment with nano-silver did not affect the weight of the chicks. The chicks in all treatment groups exhibited good viability and appearance, suggesting that the use of nano-silver did not negatively affect the overall quality of the chicks. The classification of chicks as A or B quality, based on their appearance and strength, is an important factor to consider in the poultry industry. The fact that only a small percentage of chicks (1.9%) were classified as B quality, due to weakness and dirty appearance, in this study indicates that the use of nano-silver did not have a significant negative impact on chick quality. Overall, the results of this study suggest that the use of nano-silver as a disinfectant in the poultry industry is not detrimental to the quality of chicks. The accurate measurement and assessment of chick quality is crucial for evaluating the efficacy and safety of different treatments, including the use of nano-silver. Further research is needed to investigate the long-term effects of nano-silver on chick health and growth, as well as its potential impact on the environment.

### Inductively coupled plasma analysis (ICP)

Silver ions were not detected in neither meat nor liver of the treated newly hatched chicks.

### Histopathological findings

**Fig. 4:**
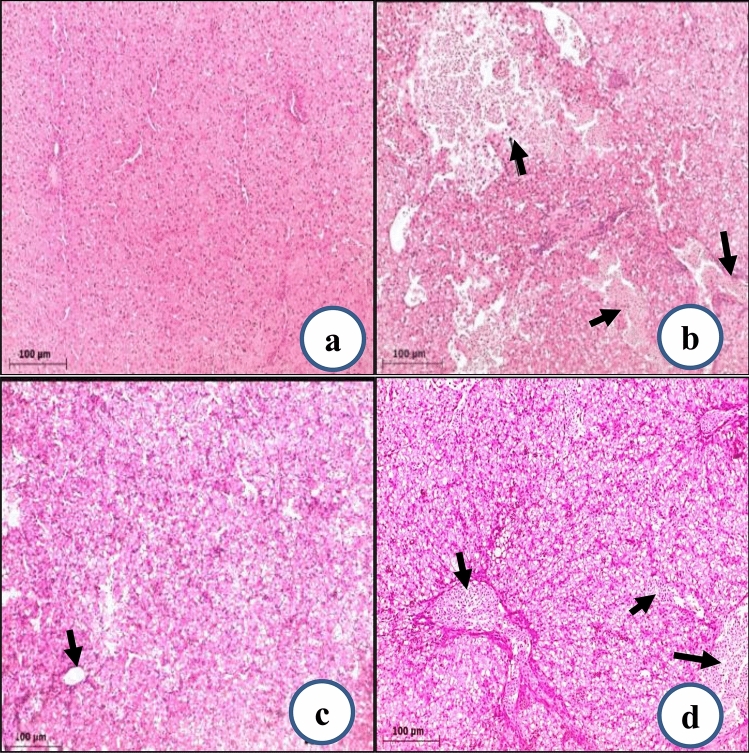
Photomicrographs of liver tissues of newly hatched chicks stained by H&E. **a** control group, **b** samples treated with bacteria-synthesized nano-silver, and **c** samples treated with plant-based synthesized nano-silver, and **d** samples treated with TH4

Histopathological evaluation of liver tissues of newly hatched chicks from untreated eggs showed normal histological characteristics with a typical hepatocyte architecture and blood vessels (Fig. 4a). In comparison, the liver tissues of the newly hatched chicks from B-Ag-NPs-treated eggs exhibited severe degeneration of hepatocytes (black arrow) (Fig. 4b). In addition, liver samples from P-Ag-NPs treated group showed moderate degeneration (Fig. 4c). Additionally, the treated samples with TH4 revealed mild degeneration of hepatocytes in the liver (Fig. 4d). It is suggested that the modifications in the liver structure could result from the penetration of potential of the tested material into eggs at early stages of embryogenesis. From the abovementioned results, it seems that B-Ag-NPs have the strongest effect on the liver. This could be regarded to its small-sized particles. Sergeevna et al. (2018) conducted research to investigate the presence of silver in the meat and organs of broiler chickens that were given colloidal silver. The study found that broiler meat contained safe levels of silver for human consumption regardless of colloidal silver usage. Furthermore, there were no significant differences in the chemical composition of the meat between the experimental and control groups. However, there were differences in the chemical composition of leg muscle and chest muscle meat. The same findings were investigated by Salem et al (2021); they detect partial residues of Ag-NPs in muscles of broiler chickens.

## Conclusion

In conclusion, the study highlights the potential of using bacterial and plant-produced silver nanoparticles (Ag-NPs) for the sanitation of hatching eggs to effectively reduce microbial load without negative effects on hatchability or embryonic mortality. However, histological analysis revealed potential hazardous effects of Ag-NPs on liver structure. Therefore, the histological changes are appropriate to be regarded as a safety parameter in Ag-NPs applications. Further research is needed to fully understand the potential risks and benefits of using Ag-NPs in sanitation practices and to explore their wider applications in various fields.
